# Deficient IL-2 Produced by Activated CD56^+^ T Cells Contributes to Impaired NK Cell-Mediated ADCC Function in Chronic HIV-1 Infection

**DOI:** 10.3389/fimmu.2019.01647

**Published:** 2019-07-16

**Authors:** Zhe Xie, Jiajia Zheng, Yuya Wang, Dan Li, Tuohutaerbieke Maermaer, Yuantao Li, Jing Tu, Qiang Xu, Hua Liang, Weiping Cai, Tao Shen

**Affiliations:** ^1^Department of Microbiology and Infectious Disease Center School of Basic Medical Sciences, Peking University, Beijing, China; ^2^Department of Laboratory Medicine, Peking University Third Hospital, Beijing, China; ^3^State Key Laboratory of Infectious Disease Prevention and Control, National Center for AIDS/STD Control and Prevention, China CDC, Collaborative Innovation Center for Diagnosis and Treatment of Infectious Diseases, Beijing, China; ^4^Department of Infectious Diseases, Guangzhou Eighth People's Hospital, Guangzhou Medical University, Guangzhou, China

**Keywords:** NK cells, CD56^+^T cells, ADCC, HIV-1, IL-2

## Abstract

**Background:** Antibody-dependent cellular cytotoxicity (ADCC), which mainly mediated by natural killer (NK) cells, may play a critical role in human immunodeficiency virus type-1 (HIV-1) disease progression. However, the potential mechanisms that affecting NK-mediated ADCC response are still not well-elucidated.

**Methods:** Antigen-antibody complex model of Ab-opsonized P815 cells was adopted to induce a typical non-specific ADCC response. The capacities of HIV-1 specific NK-ADCC were measured by using the combination model of gp120 protein and plasma of HIV-1 elite controllers. The levels of plasma cytokine were measured by ELISA. Anti-IL-2 blocking antibody was used to analyze the impact of activated CD56^+^ T cells on NK-ADCC response.

**Results:** IL-2, IL-15, IFN-α, and IFN-β could effectively enhance the non-specific and HIV-1-specific NK-ADCC responses. Compared with healthy controls, HIV-1-infected patients showed decreased plasma IL-2 levels, while no differences of plasma IFN-α, IL-15, and IFN-β were presented. IL-2 production was detected from CD56^+^ T cells activated through antibody-dependent manner. The capability of NK-ADCC could be weakened by blocking IL-2 secretion from activated CD56^+^ T cells. Although no difference of frequencies of CD56^+^ T cells was found between HIV-1-infected patients and healthy controls, deficient IL-2 secretion from activated CD56^+^ T were found in chronic HIV-1 infection.

**Conclusions:** The impaired ability of activated CD56^+^ T cells to secreting IL-2 might contribute to the attenuated NK cell-mediated ADCC function in HIV-1 infection.

## Introduction

The induction of a broad range of neutralizing antibodies and cytotoxic T lymphocyte (CTL) responses may contribute to the development of anti-HIV strategies (1), but studies have shown that non-neutralizing HIV-specific antibodies are also play an important role in preventing or controlling HIV infection. These antibodies can bind to infected cells and recruit innate immune effector cells, such as NK (natural killer) cells to clear the infected cells through antibody-dependent cellular cytotoxicity (ADCC). NK cells express CD16 (FcγRIIIa) that binds the constant (Fc) domain of IgG antibodies. CD16 engagement is a strong activator of NK cell function, and allows antigen-specific recruitment of NK responses. Over the past decades, numerous studies have confirmed that NK cells mediated ADCC (NK-ADCC) effects may play an important role in controlling HIV infection (2–4). For simian Immunodeficiency virus (SIV) infected rhesus monkeys, the animals without viremia symptoms also have more potent ADCC effect than animals with viremia (5). Importantly, ADCC activity was associated with the protective efficacy in the RV144 HIV vaccine trial (6). Moreover, some studies indicated that NK-ADCC may contribute to the elimination of the reactivated latent HIV-infected cells and then diminish the established latent reservoir (7, 8). Theoretically, it is suggested that the improved or modulated NK-ADCC response may optimize the therapeutic effect and enhance the protective anti-HIV immune responses.

Several studies have demonstrated the role of cytokines in NK cells priming. It has been reported that IL-12, IL-15, IL-18, IL-23 could augment the activation of NK cells and improve the ADCC cytotoxicity through a series of mechanisms, thus displaying important roles in anti-tumor immunotherapy or other diseases (9–11). In fact, the profiles of plasma/serum cytokines during HIV infection are complex and diverse. Studies have shown that high levels of soluble IL-10 (12), IL-7 (13), GM-CSF (14), and TNFα (15) have been observed in the plasma of HIV-1-infected patients. In addition, other studies have shown that the production of IL-12 and IL-15 were decreased in HIV-infected individuals (16, 17). However, activities of NK-mediated ADCC modulated by cytokines in HIV-1 infection are poorly understood.

In this study, we systemically evaluated the effects of different cytokines on NK cell-mediated ADCC response and screened out the cytokines that could enhance NK-ADCC response. We also evaluated serum cytokines profiles in HIV-1-infected patients and explored the potential mechanisms weakening NK-ADCC function in chronic HIV infection. Exploring how to enhance the host ADCC function will provide support for the realization of new HIV-related immune prevention and control strategies.

## Materials and Methods

### Study of Population

In our study, 50 HIV-positive patients were recruited from Guangdong No.8 People's Hospital. 30 healthy adults who had negative HIV-1 infection were recruited as healthy controls. Blood routine test, clinical biochemistries, CD4 ^+^/CD8^+^ T cells count, HIV antibody, HCV antibody, HIV RNA and HCV RNA were detected for all participants (Supplementary Table 1). All subjects have no HBV and HCV infection. Whole venous blood samples were collected from each individual. Peripheral blood mononuclear cells (PBMCs) were prepared from fresh EDTA anti-coagulated peripheral blood using Histopaque-1077 (Sigma-Aldrich, USA, 10771-500 ml) and were stored (5 × 10^6^ cells per vial) in liquid nitrogen till use. Serum and EDTA anti-coagulated plasma were stored at −80°C until use. This study was approved by the ethics committee of Peking University and informed consents were obtained from all cases.

### Pre-incubation With Cytokines of PBMCs

25 types of recombinant human cytokines (IL-2, IL-3, IL-4, IL-5, IL-6, IL-7, IL-8, IL-10, IL-12, IL-13, IL-15, IL-17A, IL-17F, IL-21, IL-22, IL-23, SCF, M-CSF, GM-CSF, G-CSF, TNF-α, IFN-α, IFN-β, IFN-γ, and IFN-λ) were purchased from PeproTech, Inc. (USA). All the lyophilized powder was reconstituted in double distilled water, sodium phosphate, acetic acid or phosphate buffer solution according to the protocol described and then diluted with PBS comprising 5% of trehalose and sub-packaged as 10 μl per tube for further use. All the dilution buffers were purchased from Multi sciences, China.

PBMCs from healthy donors were thawed and then rested overnight at 37°C. Samples were seeded as 1 × 10^5^ cell/100 μl/well in 96 well-plates. Twenty-five types of cytokines were reconstituted as 10 mg/ml and 1 μl was added into a 200 μl medium per well to construct a 50 ng/ml concentration. PBS comprising 5% of trehalose was used as negative control. Samples were incubated in a humidified CO_2_ incubator at 37°C for 12 h for further analysis.

### NK Cell-Mediated Nonspecific ADCC Activity With Stimulation of Ab-Opsonized P815 Cells

PBMCs or purified NK cells were stimulated with P815 mouse mastocytoma cells plus P815-specific Abs (1:100 dilution of polyclonal rabbit anti-mouse lymphocyte serum, Accurate Chemical & Scientific Corp., Westbury, NY) and ADCC responses were indicated through CD107a production and IFN-γ secretion from activated NK cells. P815 cells were used as target cells (18). PBMCs or purified NK cells were stimulated with P815 cells alone or Ab-opsonized P815 cells at an E: T ratio of 10:1. Then the Brefeldin-A (10 μg/ml, Sigma, St. Louis, MO, USA), GolgiStop (5 μg/ml, BD Biosciences) and anti-CD107a PE-Cy5 (clone H4A3, BD Biosciences) were added to cell medium after 1 h incubation and continued to incubate for up to 6 h in a humidified CO_2_ incubator at 37°C. Cells were stained with anti-CD3 eFluor 450 (clone UCHT1) and anti-CD56 PE-Cy7 (clone B159) and fixed by 2% PFA. All data were acquired on BD FACS Fortessa (BD Biosciences, San Jose, CA, USA) and analyzed by FlowJo software (Treestar, Ashland, OR, USA).

### Activation of NK Cells Mediated by CD16 Cross-Linking

PBMCs or purified NK cells were stimulated with 10 μg/ml of anti-CD16 antibody (clone 3G8, Santz Cruz biotechnology, Santa Cruz, CA, USA) or mouse IgG1(κ) (clone X40, BD Biosciences) isotype control for 30 min on ice. Cells were washed to remove unbound antibody and incubated with 10 μg/ml of goat anti-mouse IgG1 F(ab′)_2_ (Santz Cruz biotechnology, Santa Cruz, USA). The Brefeldin-A (10 μg/ml, Sigma, St. Louis, MO, USA), GolgiStop (5 μg/ml, BD Biosciences) and anti-CD107a PE-Cy5 (clone H4A3, BD Biosciences) were added to cell medium after 1 h incubation and continued to incubate for up to 5 h in a humidified CO_2_ incubator at 37°C. Then, cells were washed and stained with anti-CD3 eFluor 450 (clone UCHT1) and anti-CD56 PE-Cy7(clone B159) and fixed by 2% paraformaldehyde (PFA). All data were acquired on BD FACS Fortessa (BD Biosciences, San Jose, CA, USA) and analyzed by FlowJo software (Treestar, Ashland, OR, USA).

### NK Cell-Mediated Cytotoxicity Detected by Rapid Fluorometric Assay

A rapid fluorometric ADCC assay was performed as described previously (19). 1 × 10^6^ target cells were double-stained by 5 μm CFSE (Molecular Probes, Eugene, OR, USA) and 5 μm PKH-26 (Sigma, St. Louis, MO, USA). PBMCs pre-incubated with cytokines were used as the effector cells and incubated with P815 alone or Ab-opsonized P815 cells complex at an E:T ratio of 1:1. After 6 h incubation, cells were washed and fixed in cold 2% paraformaldehyde. Samples were run on BD FACS Fortessa (BD Biosciences, San Jose, CA, USA) and the abilities of NK cells killing target cells influenced by cytokines were analyzed by FlowJo software (Treestar, Ashland, OR, USA).

### Image Analysis of CD16/CD69/CD161 Expressions on NK Cells

To compare the surface expression of CD16, CD69, and CD161 on NK cells at the whole range and single-cell level, PBMCs of healthy donors were pre-incubated with cytokines for 12 h and then washed with PBS. All samples were stained with anti-CD3 eFluor 450 (ebioscience, clone UCHT1), anti-CD16 APC-Cy7 (BD, clone 3G8), anti-CD56 PE-Cy7 (BD, clone B159), anti-CD69 APC (BD, clone FN50), and anti-CD161 PE-Cy5 (BD, clone DX12). All antibodies were purchased from BD Biosciences (San Diego, CA, USA) except anti-CD3 from eBiosciences (San Diego, CA, USA). NK cells were defined as phenotype of CD3^−^CD56^+^ and the frequencies of CD16, CD69 and CD161^+^ NK cells were analyzed. CD16/CD69/CD161 expressions on NK cells were acquired on BD FACS Fortessa (BD Biosciences, USA) and then analyzed by FlowJo software (Treestar, Ashland, OR, USA).

The prepared samples were also run on ImageStream χ MarkII system (Amnis Corporation, Seattle, WA, USA) to acquire the data of CD16/CD69/CD161 expressions on single-cell. Twenty thousand events were collected for each sample and single-color control was used to create a compensation matrix to correct for spectral overlap. Collected data were analyzed using IDEAS 3.0 software (Amnis Corporation, Seattle, WA, USA).

### HIV-1-Specific ADCC Activity Assay

HIV-1_CN54_ gp120 peptides (1.0 mg/ml), purified HIV-1_CN54_ gp120 protein (1.0 mg/ml), and plasma of HIV-1 elite controllers were kindly presented by China National Center for AIDS/STD Control and Prevention. Gp120 mixed overlapping peptides or purified HIV-1_CN54_ gp120 protein were dissolved to a concentration of 1 μg/ml in PBS and were added into 96 well flat-bottom plates (Corning Costar, USA) at 4°C overnight. After washing for 3 times with 0.05% Tween 20 in PBS (PBS-T), the plates were blocked with blocking buffer (5% skim milk and 2% bovine albumin in PBS) for 1 h at room temperature (RT). Plates were then washed five times with PBS-T. 100 μl plasma of HIV-1 elite controller diluted by PBS (1:1,000) were added into each of the 96 wells and incubated at room temperature for 2 h. After washing, plates were incubated with PBMC from HIV-1-infected patients and the HIV-1-specific ADCC activity was assayed as mentioned above.

### ELISA Assay

The levels of secreted IL-2, IL-15, IFN-α, and IFN-β in serum or culture supernatants were detected by ELISA kits IL-2 (eBioscience, San Diego, CA, USA, sensitivity 2 pg/ml), IL-15 (eBioscience, sensitivity 20 pg/ml), IFN-α (Invitrogen, San Diego, CA, USA, sensitivity 3.2 pg/ml), and IFN-β (PBL assay science and assay range 50–4,000 pg/ml), according to the manufacturer's instruction.

### RNA Extraction and IL-2 mRNA Expression Analysis

Total RNA was extracted from cells by using Trizol reagent (Invitrogen, USA) according to the manufacturer's protocol. And cDNA was reverse transcribed from 3.0 μg of total RNA with random hexamer primers using a Maxima® First Strand cDNA Synthesis Kit (Fermentas, USA). qRT-PCR was performed on the LightCycler® 480IIreal-time PCR system (Roche, USA) using the QuantiTect SYBR Green PCR kit (Roche, USA). The 20 μl reaction mix contained 200 nM of each primer, 100 μl of LightCycler 480 SYBR green I master mix (Roche), and 1 μl of template cDNA. The primers for the IL-2 gene were F (5′- CAG GAT GGA GAA TTA CAG GAA CCT-3′) and R (5′-TTT CAA TTC TGT GGC CTG CTT-3′), and those used for β-actin gene were F (5′- TTG TTA CAG GAA GTC CCT TGC C-3′) and R (5′-ATG CTA TCA CCT CCC CTG TGT G-3′). For gene IL-2 and β-actin, the amplifications consisted of 10 min at 95°C followed by 40 cycles, each consisting of denaturing for 15 s at 95°C, annealing for 1 min at 60°C, and elongation for 30 s at 60°C. All PCR were performed in triplicate using cDNA synthesized from the same batch and starting amount of total RNA. The expression of IL-2 gene was determined using the comparative Ct method (2^−ΔCt^) after normalization to β-actin.

### Sorting of NK Cells

NK cells were sorted from PBMCs by EasySep Human NK cell Enrichment Kit according to the manufacturer's instruction. In brief, NK cells were negatively isolated by targeting non-NK cells (i.e., T cells, B cells, stem cells, dendritic cells, monocytes, granulocytes and erythroid cells) for removal with antibodies recognizing specific cell surface markers. Unwanted cells were bound with antibodies and magnetic particles, and separated without columns using an EasySep magnet. Desired cells are simply poured off into a new tube. The purity of NK cells obtained in this fashion was consistently >90%.

### Sorting of CD56^+^ T Depleted Lymphocytes by BD FACS AriaIII

PBMCs were stained with anti-CD3 PE (ebioscience, clone UCHT1), anti-CD56 PE-Cy7 (BD, clone B159) for 30 min at room temperature. CD56^+^ T cells were depleted by gathering CD3^+^ CD56^−^ and CD3^−^ cells on BD FACS Aria III (BD Biosciences, San Jose, CA). Only sorted samples with CD56^+^ T cells less than 5% were used in subsequent experiments.

### Evaluation of IL-2 Secretions From CD56^+^ T Cells During the Process of ADCC

The IL-2 secretions of CD56^+^ T cells, NK cells and T cells were determined by Ab-opsonized P815 cells, CD16 cross-linking, or HIV-1 gp120 protein plus HIV-1 elite controller plasma models. PBMCs were stained with anti-CD3 eFluor 450 (clone UCHT1), anti-CD56 PE-Cy7 (clone B159), anti-CD107a PE-Cy5 (clone H4A3) and anti-IL-2 FITC (clone MQ1-17H12), and fixed by 2% PFA. All data were acquired on BD FACS Fortessa (BD Biosciences, San Jose, CA, USA) and analyzed by FlowJo software (Treestar, Ashland, OR, USA).

### Blocking of IL-2 Function

IL-2 monoclonal functional antibody (AB12-3G4, eBioscience, USA) were added as 100 ng/ml/well in NK cell-mediated ADCC activity in Ab-opsonized P815 cells model and CD16 cross linking model to block IL-2 function.

### Transwell Culture Assay

Purified NK cells and CD56^+^ T cells from healthy controls (*n* = 10) were diluted in complete RPMI1640 medium containing 10% of fetal bovine serum (R10 medium) (Gibco BRL, Grand Island, NY, USA) and 1% of penicillin and streptomycin (Gbico) to the final volume of 1 × 10^6^/ml and 1 × 10^5^ cells and were seeded in the bottom wells of 96-well transwell plate (Corning Lifescience, Lowell, MA, USA). A total of four groups were set: a) NK cells alone; b) NK cells + IL-2 antibody; c) NK cells + CD56^+^ T cells (transwell); d) NK cells + CD56^+^ T cells (transwell) + IL-2 antibody. The final concentrations of NK cells, CD56^+^ T and IL-2 antibody were 1 × 10^5^/well, 1 × 10^4^/well and 100 ng/ml, respectively. Ab-opsonized P815 (1 × 10^5^/well) cells were added to all of the wells (top and bottom). After incubation for 6 h, NK cells were collected to detect degranulation with BD FACS Fortessa (BD Biosciences, San Jose, CA, USA) and then data was analyzed by FlowJo software (Treestar, Ashland, OR, USA).

### Statistical Analysis

All the statistical and graphic analyses were performed using GraphPad Prism 5.0 (GraphPad Software, La Jolla, CA, USA) or Microsoft Excel 2007. Data were expressed as mean ± SD. Comparisons between groups were performed using Mann–Whitney *U*-test, non-parametric *t*-test, or Wilcoxon matched-pairs signed rank test when necessary. All *P*-values were two-tailed and considered significant when lower than 0.05.

## Results

### Screening and Identification of Cytokines Enhancing the Non-specific NK-Mediated ADCC Responses

**Figure 1:**
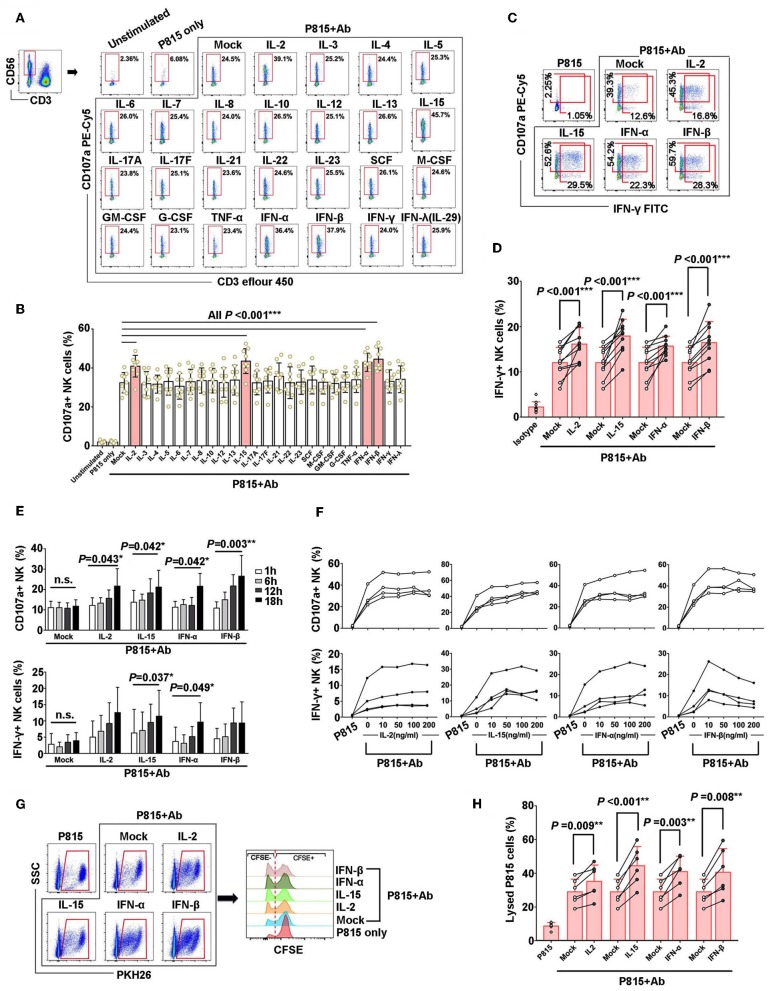
IL-2, IL-15, IFN-β, and IFN-α could augment the nonspecific NK-ADCC function. **(A)** Representative flow plots of degranulation of NK cells in response to Ab-opsonized P815 cells (P815 + Ab), or medium or P815 cells alone after pre-incubation with different cytokines (50 ng/ml) for 12 h. **(B)** IL-2, IL-15, IFN-β, and IFN-α augmented CD107a production of activated NK cells during non-specific ADCC with stimulation of Ab-opsonized P815 cells (*n* = 9). **(C)** Representative flow plots of IFN-γ secretion of NK cells after pre-incubation with IL-2, IL-15, IFN-α, and IFN-β(50 ng/ml, 12 h). **(D)** IL-2, IL-15, IFN-β, and IFN-α increased IFN-γ secretion of NK cells during non-specific ADCC with stimulation of Ab-opsonized P815 cells(*n* = 10). **(E)** Effect of pre-incubation time of IL-2, IL-15, IFN-α, and IFN-β cytokines on NK-ADCC response. CD107a expression and IFN-γ secretion of NK cells were compared among samples pre-incubation with cytokines (50 ng/ml) for different hours (1, 6, 12, 18 h) with stimulation of Ab-opsonized P815 cells (*n* = 4). **(F)** Effect of cytokine concentrations on NK-ADCC response. CD107a expression and IFN-γ secretion of NK cells were compared among samples pre-incubation with different concentrations of IL-2, IL-15, IFN-α, and IFN-β cytokines (0, 10, 50, 100, 200 ng/ml) and stimulated with Ab-opsonized P815 cells for 12 h (*n* = 4). **(G)** Representative flow plots showing the lytic abilities of NK cells after pre-incubated with IL-2, IL-15, IFN-α, IFN-β (50 ng/ml, 12 h) and co-cultured with P815 cells or Ab-opsonized P815 cells for 6 h. Target P815 cells stained with PKH26^+^ CFSE^−/low^ were indicated as lysed target cells. **(H)** Lysed rate of P815 target cells lysing by NK cells pre-incubated with IL-2, IL-15, IFN-α, or IFN-β (50 ng/ml, 12 h) and activated by Ab-opsonized cells subsequently (*n* = 6). Data is presented as mean ± SD. All *P*-values are two-tailed and considered to be significantly different with *P* < 0.05.

It has been reported that cytokines play an important role in regulation of NK cell activation and natural cytotoxicity (20, 21). In this study, firstly, healthy PBMCs were pre-incubated with 25 recombinant human cytokines, IL-2, IL-3, IL-4, IL-5, IL-6, IL-7, IL-8, IL-10, IL-12p70, IL-13, IL-15, IL-17A, IL-17F, IL-21, IL-22, IL-23, SCF, M-CSF, GM-CSF, G-CSF, TNF-α, IFN-α, IFN-β, IFN-γ, and IFN-λ, respectively. And then PBMCs were stimulated with Ab-opsonized P815 cells (P815+Ab) to evaluate the capacities of NK cell-mediated ADCC responses, which was assessed by degranulation of NK cells (indicated as CD107a production) (Figures 1A,B). Among these 25 cytokines, IL-2, IL-15, IFN-α, and IFN-β could significantly enhance CD107a expressions in NK cells with the stimulation of Ab-opsonized P815 cells (*P* < 0.001, Figures 1A,B). Similarly, IFN-γ secretion from NK cells were also significantly increased with the stimulation of Ab-opsonized P815 cells in the presence of IL-2 (*P* < 0.001), IL-15 (*P* < 0.001), IFN-α (*P* = 0.002), and IFN-β (*P* < 0.001) (Figures 1C,D). Moreover, we observed the CD107a production and IFN-γ secretion were increased as the pre-incubation time for these cytokines was extended or the concentrations of cytokines were increased (Figures 1E,F). These data suggested that the selected cytokines exerted stable and sustained effect on priming of NK cell-mediated ADCC response.

Next, to address antibody-dependent lytic capacity of NK cells, target P815 cells were pre-stained with PKH26 and CFSE, and a rapid fluorometric ADCC (RFADCC) assay was employed to detect the frequencies of CFSE^−/LOW^ target cells. When P815 cells were lysed by NK cells, the double stained P815 cells (PKH26^+^CFSE^+^) were changed as PKH26^+^CFSE^−^ (Figure 1G). Our data showed that the percentages of P815 target cells killed by NK cells were significantly elevated when pre-stimulated with IL-2 (*P* = 0.009), IL-15(*P* < 0.001), IFN-α (*P* = 0.003), and IFN-β (*P* = 0.008). Remarkably, our results suggested that IL-2, IL-15, IFN-α, IFN-β could potentiate NK cells-mediated cytotoxicity (Figure 1H).

In addition, NK cells could also be triggered by IL-2 (*P* = 0.003), IL-15 (*P* < 0.001), IFN-α (*P* < 0.001), and IFN-β (*P* = 0.002) to achieve degranulation after CD16 cross-linking with anti-CD16 monoclonal Ab (Supplementary Figure 1).

Taken together, these results confirmed that cytokines IL-2, IL-15, IFN-α, IFN-β could enhance the non-specific NK-ADCC responses.

### IL-2, IL-15, IFN-α, IFN-β Promoted CD69 Expressions on the Surface of NK Cells

**Figure 2:**
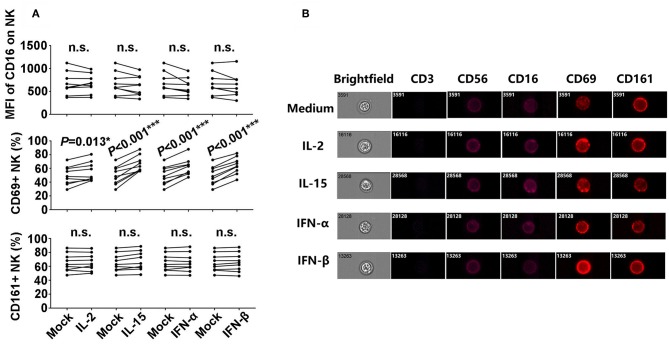
Phenotypic characteristics of NK cells after incubation with L-2, IL-15, IFN-α, and IFN-β. **(A)** Comparison of CD16, CD69, and CD161 expressions on NK cells from the healthy donors after incubation with IL-2, IL-15, IFN-α, and IFN-β (50 ng/ml, 12 h), respectively (*n* = 8). **(B)** Images of CD3, CD56, CD16, CD69, and CD161 expressions on CD56^dim^ NK cells at single-cell level after incubation with IL-2, IL-15, IFN-α, and IFN-β (50 ng/ml, 12 h), respectively. Data is acquired in an ImageStream x Mark II system. All *P*-values are two-tailed and significantly different with *P* < 0.05.

In order to explore the potential mechanisms involved in the enhancement of NK-ADCC response induced by the candidate cytokines, we detected the expressions of CD16, CD161, and CD69, which represented the activation of NK cells in the presence or absence of specified cytokines. As indicated in Figure 2A, CD69^+^ NK cells were significantly elevated after stimulation with IL-2 (*P* = 0.013), IL-15 (*P* < 0.001), IFN-α (*P* < 0.001), and IFN-β (*P* < 0.001). However, there were no significant differences in CD16 and CD161 expressions before and after cytokines stimulations. The expressions of CD16, CD161, and CD69 on CD56^dim^ NK cells were confirmed at single-cell level by image analysis (Figure 2B). CD69 showed a significantly enhanced expression upon the stimulation with cytokines (Figure 2B). CD69 is a classical marker of lymphocyte activation due to its rapid appearance on the surface of the plasma membrane after stimulation (22). Overall, IL-2, IL-15, IFN-α, and IFN-β enhanced ADCC responses through promoting the activated status of NK cells.

### IL-2, IL-15, IFN-α, IFN-β Could Augment HIV-1 Specific NK-Mediated ADCC Response

**Figure 3:**
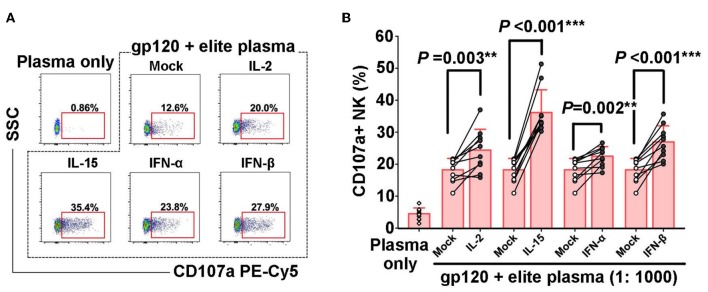
Enhancement of HIV-1-specific NK cell-mediated ADCC by cytokines. PBMCs from healthy controls (*n* = 10) were pre-incubated with IL-2, IL-15, IFN-α, or IFN-β (50 ng/ml, 12 h), respectively. CD107a expressions of NK cells in response to HIV-1 gp120 peptide pools plus plasma of elite controllers, or plasma alone were detected. **(A)** Representative flow plots of CD107a expression of NK cells in HIV-1-specific NK-ADCC response in the presence of different cytokines. **(B)** Frequencies of CD107a^+^ NK cells among different groups upon stimulation by different cytokines. Data is shown as mean ± SD. All *P*-values are two-tailed and significantly different when it is < 0.05.

Numerous studies have confirmed that ADCC plays an important role in combating HIV infection and in disease control (23). In order to further explore whether IL-2, IL-15, IFN-α, IFN-β could also enhance HIV-specific NK-ADCC response, overlapping peptides of HIV gp120 were mixed with plasma of elite controller to form HIV-specific antigen-antibody complex and then incubated with PBMCs to induce HIV-1 specific NK cell-mediated ADCC responses. It was observed that IL-2 (*P* = 0.003), IL-15 (*P* < 0.001), IFN-α (*P* = 0.002), and IFN-β (*P* < 0.001) could increase the percentages of CD107a+ NK cells (Figures 3A,B). These results suggested that IL-2, IL-15, IFN-α, IFN-β enhanced the ability of NK cells to trigger HIV-1 specific ADCC responses, which might contribute to the control of HIV infection.

### Plasma IL-2 Level Is Significantly Declined in HIV-1-Infected Patients

**Figure 4:**
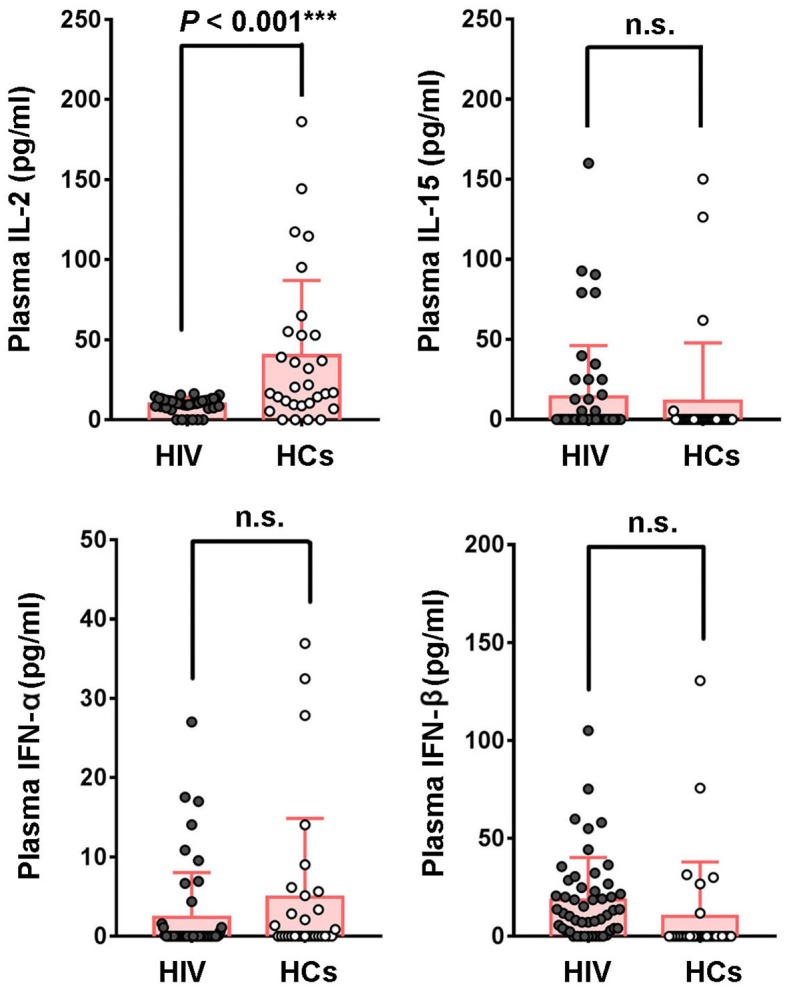
Comparison of cytokines levels in plasma between HIV-1-infected patients and healthy controls. Plasma concentrations of IL-2, IL-15, IFN-α, IFN-β were compared between HIV-1-infected patients (*n* = 50) and healthy controls (*n* = 30). Data is shown as mean ± SD. All *P*-values are two-tailed and significantly different when *P*-value is < 0.05.

Since cytokines exhibit great potential in regulating NK-mediated HIV-specific ADCC response, and the reason of impaired capacity of NK-ADCC response is still not clear, we evaluated the levels of plasma cytokines in HIV-1-infected individuals and healthy controls. There were no significant differences in plasma levels of IL-15, IFN-α and IFN-β between HIV-infected and healthy individuals. However, plasma levels of IL-2 were significantly decreased in HIV-1-infected individuals compared with healthy controls (*P* < 0.001) (Figure 4).

### CD56^+^ T Cells Could Secrete IL-2 via Antibody-Dependent Immune Response

**Figure 5:**
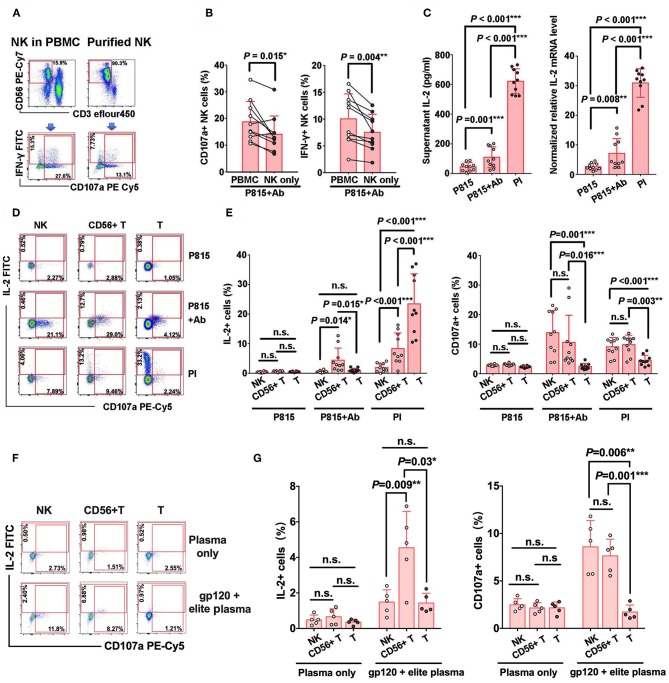
IL-2 produced by CD56^+^ T cells facilitates NK-ADCC response. **(A)** Representative flow plots of IFN-γ secretion and CD107a expression of purified NK cells and of NK cells from bulk PBMCs with the stimulation of Ab-opsonized P815 cells. **(B)** IFN-γ secretion and CD107a expression of purified NK cells were lower than that of NK cells from bulk PBMCs (*n* = 10) during ADCC response. **(C)** Secretion of IL-2 during ADCC response. PBMCs were stimulated by P815, Ab-opsonized P815 cells or PI (PMA+ionomycin) for 12 h at 37°C. Culture supernatants were collected and IL-2 level was detected by ELISA. Cell pellets were also collected and IL-2 mRNA level in PBMCs was detected by qRT-PCR (*n* = 10). **(D)** Representative flow plots of IL-2 and CD107a expressions on CD56^−^ T cells, CD56^+^ T cells and NK cells with the stimulation of P815 cells only, Ab-opsonized P815 cells or PI. **(E)** Comparison of the abilities of IL-2 secretion and CD107a expression among CD56^−^ T cells, CD56^+^ T cells and NK cells with different stimulators (*n* = 10). **(F)** Representative flow plots of IL-2 and CD107a expressions on CD56^−^ T cells, CD56^+^ T cells and NK cells with the HIV-1 elite plasma in the presence or absence of gp120 protein antigen. **(G)** Comparison of the abilities of IL-2 secretion and CD107a expression among CD56^−^ T cells, CD56^+^ T cells and NK cells with different stimulators (*n* = 5). All *P*-values are two-tailed and significantly different when the value is <0.05.

In order to explore the potential mechanisms that might affect NK-ADCC activity, we compared the cellular degranulation and IFN-γ secretion between purified NK cells and NK cells in bulk PBMCs. Our results revealed lower expressions of CD107a (*P* = 0.015) and IFN-γ (*P* = 0.004) in purified NK cells compared with NK cells in PBMCs after co-cultured with Ab-opsonized P815 cells (Figures 5A,B), suggesting that some other cells in PMBCs may play an auxiliary role in NK-ADCC process.

Considering that IL-2 could augment NK-mediated ADCC responses *in vitro* and that lower levels of plasma IL-2 were presented in HIV-1-infected patients, we examined whether IL-2 production existed in bulk PBMC during NK-ADCC response. As speculated, with the stimulation of Ab-opsonized P815 cells, IL-2 production was significantly elevated both in protein level (*P* = 0.001) as detected in supernatants and in mRNA level (*P* = 0.008) as detected in cell pellets (Figure 5C). Our previous results demonstrated that peripheral blood CD56^+^ T cells were capable to mediate antibody-dependent response (19). Four outlier individuals in P815+Ab group in Figure 5C were found to have relatively higher frequencies of CD56^+^ T cells but not CD16 expression on CD56^+^ T cells and frequencies of NK cell and its subsets, compared with that of other individuals (data not shown). Furthermore, we found that CD56^+^ T cells could also produce IL-2 via antibody-dependent activation during NK-ADCC progress. As shown in Figures 5D,E, and Supplementary Figure 2, CD56^+^ T cells in PBMCs produced much higher levels of IL-2 than conventional T cells (CD56^−^ T) (*P* = 0.015) and NK cells (*P* = 0.014) with the stimulation of Ab-opsonized P815 cells. In contrast, CD56^−^ T cells produced higher levels of IL-2 than NK cells (*P* < 0.001) and CD56^+^ T cells (*P* < 0.001) with the stimulation of PMA plus Ionomycin (PI). More importantly, similar results were observed in the HIV-1-specific ADCC and CD16 cross-linking system, that CD56^+^ T cells also produced much higher levels of IL-2 than CD56^−^ T cells (HIV: *P* = 0.03, CD16 cross linking: *P* < 0.001) and NK cells (HIV: *P* = 0.009, CD16 cross linking: *P* < 0.001) (Figures 5F,G and Supplementary Figures 3A,B). These results indicated that CD56^+^ T cells can produce IL-2 during NK-ADCC responses. These results indicated that CD56^+^ T cells can produce IL-2 during NK-ADCC responses.

### Dysfunction of CD56^+^ T Cells Contributes to the Impaired NK-ADCC in Chronic HIV-1 Infection

**Figure 6:**
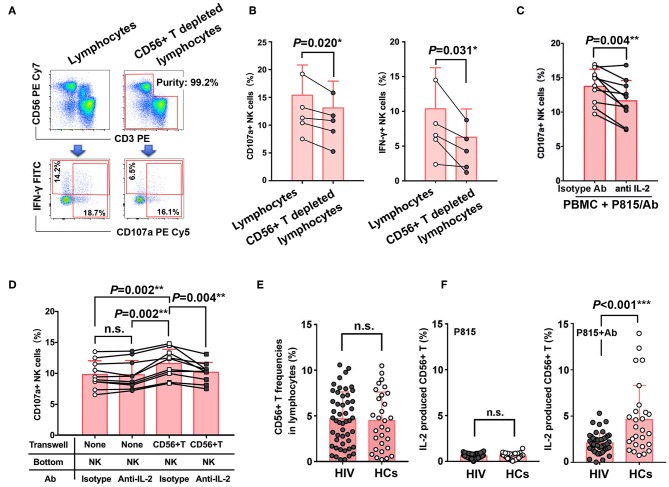
Dysfunction of CD56^+^ T cells contributes to the impaired NK-ADCC response in chronic HIV subjects. **(A)** Representative flow plots of lymphocytes in PBMC and CD56^+^ T depleting lymphocytes sorted by BD FACS Aria III. **(B)** Comparison of the abilities of CD107a and IFN-γ expression of NK cells from lymphocytes in PBMC and CD56^+^ T depleting lymphocytes (*n* = 5). **(C)** Comparison of CD107a expression of NK cells in the present or absent of anti-IL-2 antibody during the process of ADCC response. ADCC response is triggered by Ab-opsonized P815 cells (*n* = 10). **(D)** Purified NK cells were cultured with or without IL-2 antibody and/or CD56^+^ T cells (transwell insert) (*n* = 10). Ab-opsonized P815 cells were added to all wells (top and bottom) to trigger the ADCC response. The frequencies of CD107a+ NK cells between groups were compared. **(E)** Comparison of frequencies of CD56+ T cells in lymphocytes between HIV-1-infected patients (*n* = 50) and healthy controls (*n* = 30). **(F)** Comparison of the abilities of IL-2 secretion from CD56^+^ T between HIV-1-infected patients (*n* = 50) and healthy controls (*n* = 30), with the stimulations of Ab-opsonized P815 cells or P815 cells alone. Data is shown as mean ± SD. All *P*-values are two-tailed and significantly different when *P*-value is < 0.05.

To further confirm that IL-2 secreted by CD56^+^ T cells during NK-ADCC response contributes to ADCC effect of NK cells, we sorted lymphocytes and CD56^+^ T depleted lymphocytes by BD FACS Aria III from PBMC and NK-mediated ADCC were then detected in the stimulations of Ab-opsonized P815 cells (Figure 6A). As shown in Figure 6B, lower CD107a production (*P* = 0.020) and weaker IFN-γ secretion (*P* = 0.031) were found in NK cells from CD56^+^ T depleted lymphocytes than from intact lymphocytes (Figure 6B). Furthermore, IL-2 functional blocking antibody was added to block the production of IL-2 during ADCC effect. The cellular degranulation of NK cells was weakened after IL-2 blockade either in the stimulations of Ab-opsonized P815 cells (*P* = 0.004) (Figure 6C) or CD16 cross-linking model (*P* = 0.004) (Supplementary Figure 4), indicating that IL-2 was involved in the process of NK-ADCC response. Furthermore, transwell experiment indicated that NK cells showed a stronger degranulation ability (*P* = 0.002) when purified CD56^+^ T cells were placed in the transwell insert, while the ability was decreased after IL-2 functional antibodies were added (*P* = 0.004) (Figure 6D). Taken together, our results demonstrated that IL-2 derived from activated CD56^+^ T cells could facilitate NK cell-mediated ADCC responses.

In order to figure out whether CD56^+^ T cells were involved into the impaired NK-ADCC function in HIV-1 infection, we compared the frequencies of CD56^+^ T cells between HIV-1-infected patients and healthy controls and no significant difference was found (Figure 6E). However, a significantly higher IL-2 secretion by CD56^+^ T cells from healthy controls than from HIV-1-infected patients (*P* < 0.001) was observed upon stimulation with Ab-opsonized P815 cells (Figure 6F). The impaired abilities of CD56^+^ T cells to secret IL-2 may contribute to weaken NK cell-mediated ADCC function in HIV-1 infection.

## Discussion

NK cells play a key role in immune response against HIV infection. Increased NK cell activity, reflecting by higher cytotoxic capacity, IFN-γ and chemokines production, has been associated with resistance to HIV infection and delayed AIDS progression, indicating the significance of NK cells in antiviral response. So far, there are few systematic studies about screening and identification of cytokines which can enhance NK-mediated ADCC response. In this study, we found IL-2, IL-15, IFN-α, and IFN-β could significantly augment NK cell-mediated non-specific and HIV-1 specific ADCC responses. As a T-cell growth factor, IL-2 is predominantly secreted by CD4^+^ T cells to regulate the differentiation and proliferation of lymphocytes. IL-2 production declines and IL-2-producing T cells is impaired in chronically HIV-1-infected patients, as indicated by loss of CD4^+^ T cells and progression to AIDS (24). The decreased plasma IL-2 levels were also observed in our HIV-1-infected patients compared with healthy controls. Numerous studies have shown that ART and *in vivo* administration of IL-2 could partially restore NK cell distribution and function in HIV-1 infection (25, 26). Moreover, in our study, we found that CD56^+^T cells could produce much higher levels of IL-2 compared with conventional T cells during NK-ADCC process in the stimulations of Ab-opsonized P815 cells or by CD16-cross linking as shown in the Figure 5D and Supplementary Figures 1, 2. As excepted, conventional T cells could produce much higher levels of IL-2 than CD56^+^ T cells in the stimulation of PMA plus Ionomycin (Figure 5D). In despite of the absolute numbers of conventional CD3^+^T cells and its role in IL-2 production, conventional T cells could not secrete enough IL-2 in a Fc-receptor dependent manner. Taken together, we demonstrated that activated CD56^+^ T cells could produce IL-2 through the antibody-Fc-dependent manner and thus facilitate NK-ADCC response during HIV infection.

Besides, several studies have demonstrated that IL-15 and IFN-α could enhance NK cell-mediated cytotoxicity in HIV-1-infected subjects through increasing chemokines production, inhibiting viral entry to CD4^+^T cells and limiting its spread (27, 28). However, we failed to observe any differences of peripheral levels of IL-15 and IFN-α between HIV-1-infected patients and healthy controls in our study. Actually, there are also other cytokines which were not mentioned in our study but still could augment NK cells functions. A study have reported that IL-18 could augment IFN-γ production and ADCC function of NK cells activated through Fc receptors *in vitro*, and could synergy with IL-2, IL-15, and IL-21 to potentiates NK cell effector function during innate and adaptive immune responses (29). IL-27 acts as a pro-inflammatory cytokine that, in concert with other DC-derived cytokines, hierarchically contributes to NK cells activation and effector functions (30). It is unfortunately that IL-18 and IL-27 were not included in our present study. We will further investigate the synergistic effect of IL-18/IL-27 and other cytokines such as IL2/IL15/IFNα/IFNβ on NK cells ADCC functions in our following research.

Many studies reported the levels of cytokines in healthy individuals and HIV or HBV or HCV infected patients (31, 32). However, the ranges of many cytokines did not show much consistency across different recruited cohorts. In addition, to some extent, different detecting reagent kits may influence its consistency. Usually, the intense early cytokine storm occurs in acute HIV-1 infection. While, the decreased percentage of cells expressing IL-2 and serum IL-2 level were found in chronic and progressive HIV infection (33–35), which is consistent with our data. In our study, there are great differences in cytokine levels among different healthy individuals. The levels of most of these four cytokines (IL-2, IL-15, IFN-α, and IFN-β) are very low, even below the detection limit, while higher values were only found in very few individuals.

**Figure 7:**
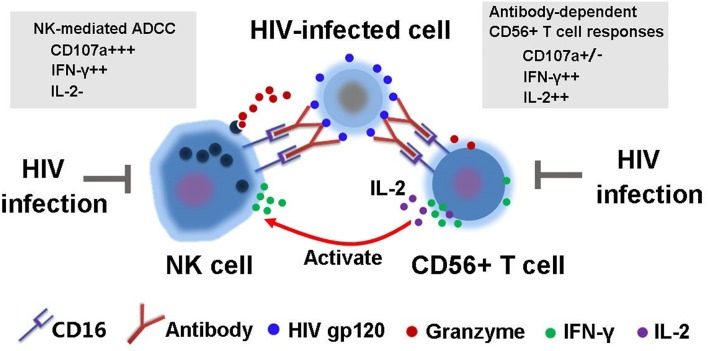
Schematic diagram of interaction between NK cells and CD56^+^ T cells during the process of HIV-1-specific ADCC immune response.

In addition, our experiments demonstrated that the ability of purified NK cells to mediate ADCC effects were significantly weakened compared to NK cells from bulk PBMCs, suggesting that other cells in PBMCs may facilitate NK-mediated ADCC. A number of studies have demonstrated that cell-to-cell interactions are important for the immune responses of NK cells against viral infections and malignancies (36–38). CD56^+^ T cells, also termed as CD3^+^CD56^+^ NKT-like cells (39, 40), account for 5% to 15% of peripheral blood T-cell pool and are considered as a superior latent source of IFN-γ (19, 41). Most CD3^+^CD56^+^ T cells express TCR αβ and only a fraction of them express TCR γδ chain (42). Recent study from our lab also confirmed that CD56^+^ T cells can mediate antibody-dependent responses through CD16, and the impaired immune activities mediated by CD56^+^ T cells were observed in long-term HIV-1-infected patients (19). On the other hand, the characteristics of CD56^+^ T cell-mediated antibody-dependent responses were different from ADCC activities mediated by classic NK cells. NK-mediated ADCC was marked by both powerful degranulation and IFN-γ production while degranulation capacity was significantly weaker in CD56 ^+^T cell-mediated antibody-dependent response though a comparable level of IFN-γ production was observed (Figure 7) (19). Currently, in this study, we demonstrated that CD56^+^ T cells could also secret IL-2 during the process of NK-ADCC and the ability was impaired in HIV infection. The decreased level of plasma IL-2 in HIV-1-infected individuals may be one of the critical reasons for the weakened ADCC activities, indicating that IL-2 produced by activated CD56^+^ T cells may actually facilitate NK cell-mediated ADCC responses.

CD56^+^ T is a mixed cell population, composing of many different cell types and might including a partial of mucosal associated invariant T cell (MAIT) and γδ^+^ T cells. Our previous work analyzed the phenotypic characteristics of CD56^+^ T cells according to the CD4 and CD8 expressions (19). We demonstrated a decreased frequency of CD4^+^CD56^+^ T cells but increased frequency of CD8^+^CD56^+^ T cells in HIV-1 infection. However, CD8^+^CD56^+^ T subset showed less capacity to mediate ADCC compared with DN (CD4^−^CD8^−^) subset. A proportion of human MAIT cells in peripheral blood express CD56, and CD56^+^ MAIT cells may have a higher capacity to respond to IL-12 and IL-18 (42). However, it has not been reported whether MAIT has ADCC or Fc receptor-dependent activation. Although some of γδ^+^ T cells were reported to express CD16 and have the ability to mediate ADCC function (43, 44), we still confused whether CD56^+^ γδ^+^ T cells express CD16 and have the ability to mediate ADCC function or not. Our subsequent studies will be interested in studying the potential capacity and mechanism of Fc dependent activation of CD56^+^ MAIT cells and γδ^+^ CD56^+^ T cells, and its potential significance in viral infections such as HIV.

In summary, the present study demonstrated that cytokines IL-2, IL-15, IFN-α, IFN-β can enhance the ADCC effect of NK cells *in vitro*. Also, we provided a novel mechanism that IL-2 secreted by activated CD56^+^ T through antibody-dependent manner can enhance the capabilities of NK cells to mediate ADCC response. Therapeutic strategy of promoting IL-2 secretion by activating CD56^+^ T cells *in vivo* to enhance NK-mediated ADCC activities may bode well for the HIV immunotherapy in the future.

## Data Availability

All datasets generated for this study are included in the manuscript and the Supplementary Files.

## Ethics Statement

This study was carried out in accordance with the recommendations of the ethical committee of our Institution (Peking University, China) with written informed consent from all subjects. All subjects gave written informed consent in accordance with the Declaration of Helsinki. The protocol was approved by the ethics committee of Peking University.

## Author Contributions

TS, JZ, and HL designed, analyzed and provided overall guidance for the experiments. ZX, YW, TM, DL, JT, QX, and YL performed the experiments. WC provided the clinical HIV-1-infected samples. JZ, ZX, and TS wrote the manuscript. All authors read and approved the final manuscript.

### Conflict of Interest Statement

The authors declare that the research was conducted in the absence of any commercial or financial relationships that could be construed as a potential conflict of interest.
