# Supplementary material for: Deficient IL-2 Produced by Activated CD56+ T Cells Contributes to Impaired NK Cell-Mediated ADCC Function in Chronic HIV-1 Infection
Source: Front Immunol. 2019 Jul 16;10:1647. doi: 10.3389/fimmu.2019.01647 (PMC6648879; doi:10.3389/fimmu.2019.01647)
Supplement: Supplementary file 1 [file Data_Sheet_1.docx]

Supplementary Material

# Supplementary Figures and Tables

## Supplementary table 1.

**Supplementary table 1.** Characteristics of HIV-1-infected patients and controls.

| Characteristics | HIV-1 infectious | Healthy |
| --- | --- | --- |
| Number | 50 | 30 |
| Sex ratio^a^ | 42/8 | 25/5 |
| Age (years)^b^ | 40.18 (13.9) | 35.70 (13.2) |
| Anti-HIV | Positive | Negative |
| CD4 cells/μl^b^ | 307.22(251) | NA^C^ |
| CD8 cells/μl^b^ | 598.70 (359) | NA |
| HBsAg | Negative | Negative |
| Anti-HCV | Negative | Negative |
| HCV RNA | Negative | Negative |
| HAART^d^, n (%) |  |  |
| Occasional | 3(6) | NA |
| Intermittent | 5(10) | NA |
| Regular | 41(82) | NA |
| Unclear | 1(2) | NA |
| Duration, median years (IQR^e^) | 8.5(6.5-10.4) | NA |

^a^ Sex ratio (male/female); ^b^ Mean ± standard deviation (SD); ^c^ NA: Not applicable. ^d^ The first-line highly active antiretroviral therapy (HAART) regimes consisting of two nucleoside reverse transcriptase inhibitors (NRTIs) AZT/ddI (~60%) or d4T/3TC (~40%), and one NNRTIs NVP, which were supported by the China CARES (Community AIDS Resource and Education Services) program; ^e^IQR, Interquartile range.

## Supplementary Figures

**
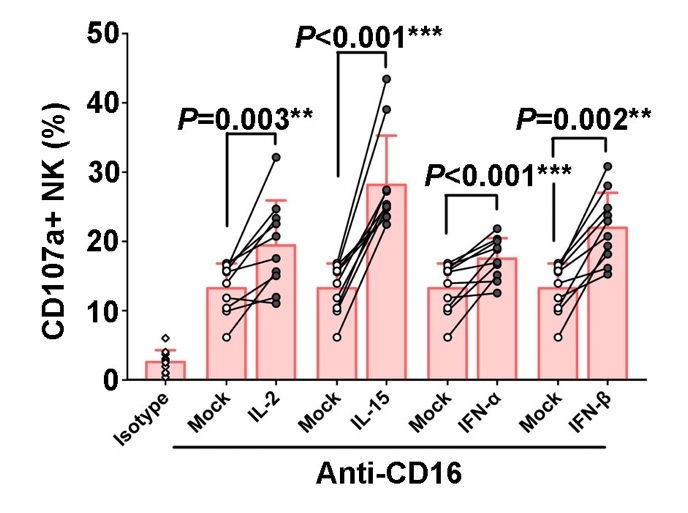
**

**Supplementary Figure 1.** Activation of nonspecific NK cell-mediated ADCC by CD16 cross-linking upon the stimulation of different cytokines. PBMCs in healthy controls (n=10) were incubated with IL-2, IL-15, IFN-α or IFN-β (50ng/ml, 12h). CD107a expression of NK cells in response to CD16 cross-linking with anti-CD16, or isotype antibodies were detected. Data is shown as mean+ SD. All P values are two-tailed and significantly different with P less than 0.05.

**
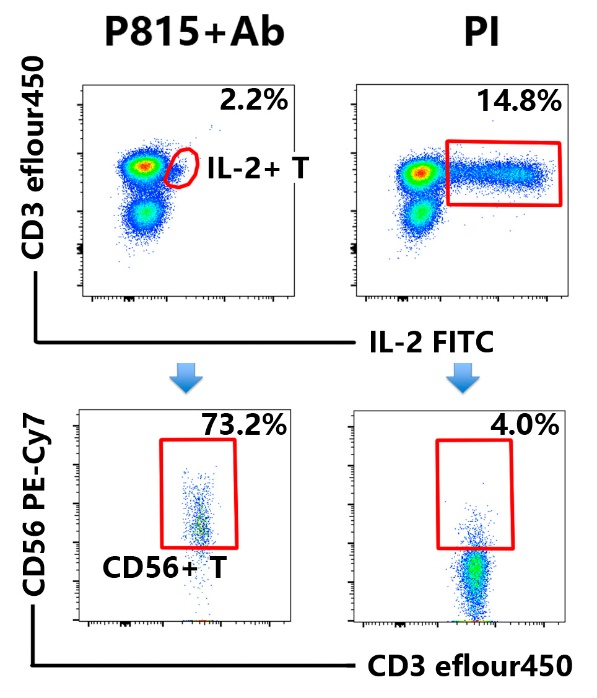
**

**Supplementary Figure 2.** Representative flow plots of IL-2 expression cells of CD56+T cells during the process of ADCC triggered by Ab-opsonized P815 cells (P815+Ab) and with the stimulation of PMA plus Ionomycin (PI).

**
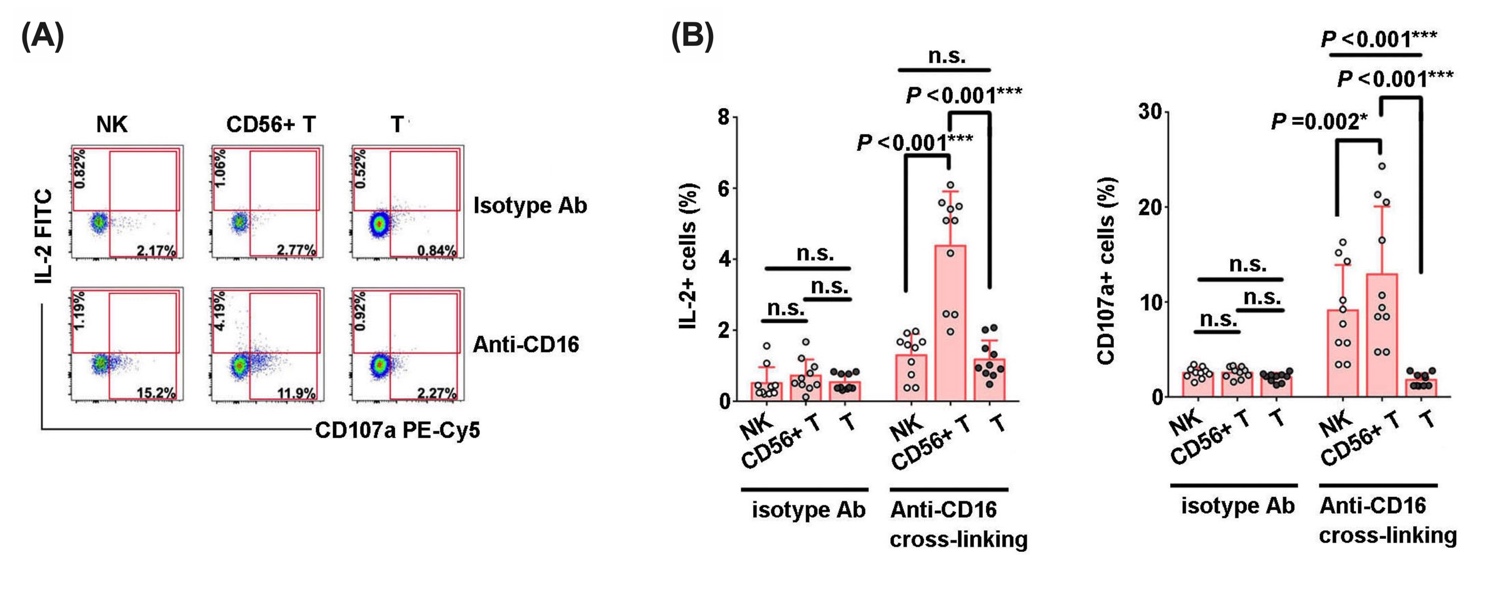
**

**Supplementary Figure 3**. **(A)** Representative flow plots of IL-2 and CD107a expressions in T cells, CD56^+^ T cells and NK cells that were triggered by CD16-cross linking. **(B)** Comparison of the abilities of IL-2 secretions and CD107a expressions among NK cells, CD56^+^ T cells and T cells that were triggered by CD16-cross linking (n=10). Data is shown as mean+ SD. All P values are two-tailed and significantly different when the value is less than 0.05.

**
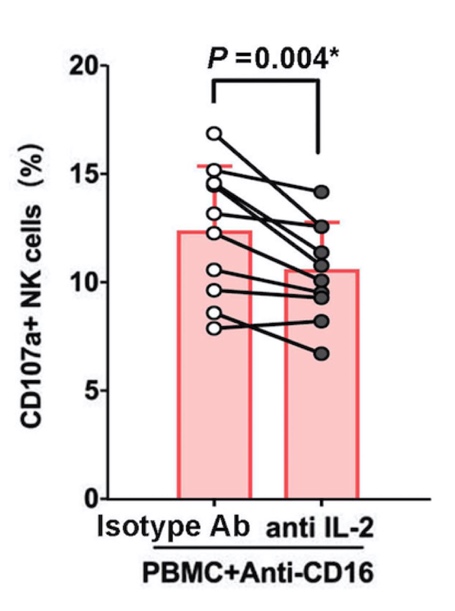
**

**Supplementary Figure 4.** Comparison of CD107a expression in NK cells triggered by CD16 cross-linking with or without blockade of IL-2 antibody(n=10).
